# Postpartum women’s use of medicines and breastfeeding practices: a systematic review

**DOI:** 10.1186/s13006-015-0053-6

**Published:** 2015-10-28

**Authors:** Moni R. Saha, Kath Ryan, Lisa H. Amir

**Affiliations:** Judith Lumley Centre, La Trobe University, 215 Franklin St, Melbourne, Vic 3000 Australia; School of Nursing and Midwifery, La Trobe University, Bundoora, Vic 3086 Australia

**Keywords:** Breastfeeding, Lactation, Maternal, Medicine, Medication, Postpartum

## Abstract

The objectives of this article are to systematically review i) the extent of medicine use in postpartum women, and ii) the impact of maternal medicine use (excluding contraceptives and galactogogues) on breastfeeding outcomes (initiation and/or duration). PubMed, Medline (Ovid), Scopus (Elsevier), Cinahl (EBSCO), PsycINFO (Ovid), Embase (Ovid) and Web of Science (ISI) databases were searched to find original studies on medicine use in women after the birth. Additional studies were identified by searching Google Scholar, Wiley Online Library, Springer Link, selected journals and from the reference list of retrieved articles. Observational studies with information about postpartum women’s use of any type of medicine either for chronic or acute illnesses with or without breastfeeding information were included. The majority of relevant studies suggest that more than 50 % of postpartum women (breastfeeding or not) required at least one medicine. Due to the lack of uniform medication use reporting system and differences in study designs, settings and samples, the proportion of medicine use by postpartum women varies widely, from 34 to 100 %. Regarding the impact of postpartum women’s medicine use on breastfeeding, a few studies suggest that women’s use of certain medicines (e.g. antiepileptics, propylthiouracil, antibiotics) during lactation can reduce initiation and/ or duration of breastfeeding. These studies are limited by small sample size, and with one exception, all were conducted in Canada more than a decade ago. Large scale studies are required to establish the relationship between maternal medicine use and breastfeeding, considering type of illness, period of use and total duration of medicine use.

**Electronic supplementary material:**

The online version of this article (doi:10.1186/s13006-015-0053-6) contains supplementary material, which is available to authorized users.

## Introduction

During the postpartum period, many women face acute and chronic health problems such as cough/colds, infections, bowel problems, mastitis, headache, back pain, migraine, hypertension, depression [1–7], and need to take medication. Fortunately, most commonly used medicines are considered safe during lactation and adverse effects of maternal medicine use on breastfed infants are rare or minimal [8–10]. The need to take medicine, however, is one of women’s self-reported reasons for discontinuation of breastfeeding [2, 11, 12].

In addition, many health professionals lack evidence-based knowledge and may advise women to stop breastfeeding while taking medicines [13–17]. A critical review of health professionals’ knowledge, attitudes and practices towards medicine use in breastfeeding women has highlighted this issue [18].

An USA study about health professionals’ knowledge of women’s issues and epilepsy showed that more than 50 % of health professionals did not know that women taking most antiepileptics could breastfeed safely [19]. In addition, a study conducted in Puerto Rico revealed that 39 % of physicians thought breastfeeding should be contraindicated for women using antidepressants [20]. Australian studies have shown that about one third of general practitioners and community pharmacists were not aware that ibuprofen is compatible with breastfeeding [21, 22].

In addition, the reliability of safety information for commonly used medicines in information databases (e.g. the Physicians’ Desk Reference) is not always accurate [23], which results in many women being inappropriately advised to stop breastfeeding. Moreover, studies in breastfeeding women and their infants are rarely conducted and clinical risk assessment for many drugs required by breastfeeding women is often compromised due to lack of data [24].

Despite breastfeeding being actively promoted, the issue of women’s use of medicines has not received much attention [25]. The extent of maternal use of medicine during the postpartum period has not been reviewed thoroughly and the impact of postpartum maternal medicine on initiation and duration of breastfeeding is not established. Therefore, the objectives of this paper are to systematically review the literature about the extent of medicine use in postpartum women, and evaluate whether or not there is a negative impact of maternal medicine use postpartum on breastfeeding outcomes especially on initiation and /or duration of breastfeeding.

## Review

### Selection criteria of articles

#### Inclusion criteria

Any English language full reports of original studies about medicine use for acute or chronic illnesses in postpartum women with or without breastfeeding information. Articles in languages other than English meeting the above criteria were also selected.

#### Exclusion criteria

Studies based on contraceptives and galactogogues use only were excluded because of their potential to modulate maternal milk supply and are not the subject of our review.

### Literature search, study selection and screening process

We conducted the primary literature search in PubMed, Medline (Ovid), Scopus (Elsevier), Cinahl (EBSCO), PsycINFO (Ovid), Embase (Ovid) and Web of Science (ISI) databases  using ‘medications’, ‘medicines’, ‘drug therapy’ ‘maternal’, ‘women’, ‘mothers’, ‘postpartum’, ‘postnatal’, lactation’ and ‘breastfeeding’ as search terms in different combinations since the start of each database. No specific limitation was applied while searching. The literature search was conducted in November 2012 to March 2013. An update of the search was conducted in August 2015.

We conducted a secondary literature search in Google Scholar, Wiley Online Library, Springer Link and selected journals (*Pediatrics*, *Pharmacoepidemiology and Drug Safety*, *Breastfeeding Medicine*, *Journal of Human Lactation* and *International Breastfeeding Journal*) to find relevant article titles using some selected therapeutic group names or selected medicine names and breastfeeding as key words. The name of therapeutic groups included in the search were ‘antidepressants or SSRIs or selective serotonin reuptake inhibitors’, ‘antipsychotics’, ‘antihypertensives’, ‘antiasthmatics’, ‘thyroid medications’, ‘antimigraine medications’ and ‘antiepileptics’. Selected medicines used in the search were ‘paroxetine’, ‘sertraline’, ‘citalopram’, ‘escitalopram’ and ‘fluoxetine’. The objective of using these selective therapeutic groups or medicines in the search was to find any studies about these specific groups or medicines which were not identified in the primary search. The reference lists of retrieved articles were also checked and previously unidentified studies located whenever possible.

After removal of duplicates, irrelevant articles were eliminated by screening the titles and/or abstracts. Then the potential full text articles were assessed for eligibility against selection criteria described earlier. MRS conducted the literature search and screened the articles in consultation with KR and LHA.

### Quality assessment of the articles

The selected full text research articles were studied thoroughly and the quality of each eligible article was assessed independently by MRS and LHA using the checklist adopted by Macfarlane et al. [26] designed from Downs and Black [27] and Crombie [28]. The checklist contained 8 items for abstracts and 20 items for full articles for cross-sectional studies or 22 items for cohort studies (Additional file 1). Each item in the checklist was scored as ‘yes’, ‘no’ or unable to determine where there was insufficient or unclear information. Each positively scored criterion was added for both abstract and full-text article separately to obtain a total quality score, expressed as a percentage, per study (adding the number of ‘yes’ per item; abstract and paper: 22 items for cohort study, and 20 items for cross-sectional study). The average score per item in the checklist for the total number of studies was also calculated adding the number of ‘yes’ per item and was expressed as a percentage. Multiple articles reporting the same study were assessed separately and an average score was reported for that study.

In addition, the quality of eligible cohort studies reporting the impact of maternal medicine use on breastfeeding was assessed using the Newcastle-Ottawa quality assessment scale [29] in which a study can be awarded a total of 9 stars for 8 items: 4 items for ‘selection’, 1 item for ‘comparability’ and 3 items for ‘outcome’ (Additional file 2). A maximum of one star can be given for each numbered item within the ‘selection’ and ‘outcome’ categories and maximum of two stars for ‘comparability’. Any disagreements about the methodological quality of the articles were resolved in a discussion between team members. In addition, weakness and bias of the studies were also noted.

### Data extraction

First, we divided all the included articles into two groups: i) studies which were focused on different types of medicine use with or without information about breastfeeding practices and ii) studies which analysed the impact of medicine use (group or specific) on breastfeeding statistically. Then, we extracted the data in tabular form which included author, publication year, country of study, study type, outcome measures, most frequently used medicines, breastfeeding information and findings of statistical analyses, quality assessment score and limitation(s). MRS synthesized the data which was also checked by LHA for accuracy. During data synthesis two primary outcome measures were abstracted: 1) extent of medicine use in postpartum women which was defined as the proportion (%) of women (breastfeeding or not) using one or more medicines and 2) impact of postpartum women’s use of medicine (any group or specific) on initiation and/or duration of breastfeeding. We also abstracted three secondary outcomes where available: 1) most frequently used medicines by postpartum women, 2) comparison of types of medicine used by postpartum women in different countries according to the Anatomical Therapeutic Chemical (ATC) classification system developed by the World Health Organization (WHO) which is recognized as the international language and gold standard for drug utilization research and 3) women’s concerns, behaviour and decision-making about use of medicines while breastfeeding. We summarized the findings in tables according to the chronological order of publication year of the articles.

## Results

### Study selection

**Fig. 1:**
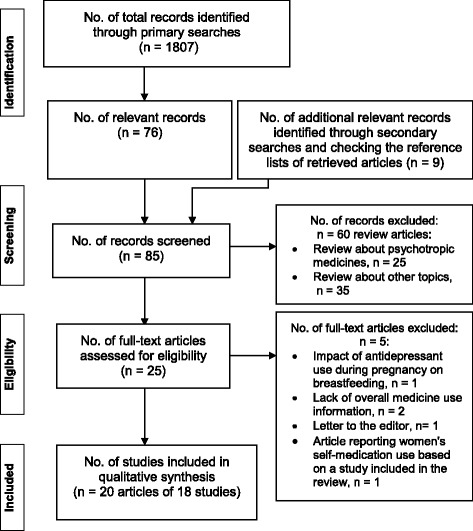
Steps of study selection process using a PRISMA flow diagram [30]

Figure 1 illustrates the study selection process using a PRISMA flow diagram [30].

From the primary literature searches we found 1807 citations. First, we removed 390 duplicate citations and then 1341 were removed after checking the titles and/or abstracts leaving 76 relevant articles. Our secondary literature searches and checking the reference lists of retrieved articles resulted in additional 9 relevant articles for screening. Thus we screened 85 articles for eligibility assessment. Review articles were eliminated (*n* = 60; 25 about safety and compatibility of psychotropic medicines while breastfeeding, and 35 other topics like oral contraceptive use and breastfeeding and others). Finally, 25 full-text articles [31–55] (24 English and 1 Portuguese [42]) were assessed for eligibility for inclusion. Five further articles were excluded: one article based on impact of antidepressant use during pregnancy on breastfeeding [32]; two articles did not contain overall medicine use information [31, 40]; one article [35] contained information about maternal use of self-medication only and was based on the same cohort used in another article [33] included in this review; and another one [54] was a letter to the editor. Two articles [45, 46] using the same cohort were considered as a single study and two articles [41, 44] from the same survey were also abstracted as one study. Thus, a total of 20 articles [33, 34, 36–39, 41–53, 55] consisting of 18 studies were included in the review. The update of the literature search in August 2015 did not identify any new primary studies for inclusion.

### Study details

Of the 18 selected studies, 10 consisting of 11 articles [33, 34, 36–38, 43, 45–48, 51] were cohort studies and 8 consisting of 9 articles [39, 41, 42, 44, 49, 50, 52, 53, 55] were cross-sectional in design. The studies are summarized in Tables 1 and 2.

**Table 1:** Extent of medicine use in women during postpartum period or lactation

Author and publication year	Country, study type, sample, design and settings	Postpartum/lactation period	Proportion of women using ≥ 1 medicine during lactation and frequently used medicines	Macfarlane’s quality score, and limitation(s)
Chaves et al., 2011 [33]	Brazil, cohort study, *n* = 246	12 months	98 %	Quality score: Abstract: 88 %, Paper: 77 %
	Pregnant women were recruited between June and Sept 2003. Nursing mothers were followed up after hospital discharge by telephone contact or home visits up to 12 months postpartum or until breastfeeding ceased.		Analgesics/antipyretics 24.7 %	
			Iron salts 13.7 %	Limitation(s): Commonly used medicines were reported as % of total number of prescriptions. ATC classification was not used.
			NSAIDs 12.8 %	
			(% based on total number of prescriptions)	
Stephansson et al., 2011 [34]	Sweden, cohort study, *n* = 102,995	3 months	51 %	Quality score: Abstract: 75 %, Paper: 64 %
	Women using prescription medicines during pregnancy and 3 months postpartum in 2007, collected from Swedish Medical Birth Register and the Prescribed Drug Register.		Sex hormones 21.91 %	
			Systemic antibacterials 13.77 %	Limitation(s): Actual drug intake bias as data source was register. No breastfeeding information was available
			NSAIDs 7.06 %	
Engeland et al., 2008 [36]	Norway, cohort study, *n* = 106,329	3 months	57 %	Quality score: Abstract: 75 %, Paper: 64 %
	Women using prescription medicine during pregnancy and 3 months postpartum, collected from Medical Birth Registry of Norway and Norwegian Prescription Database of 2004–2006.		Sex hormones 26.7 %	
			Systemic antibacterials 16 %	Limitation(s): Actual drug intake bias as data source was register. No breastfeeding information was available
			Posterior pituitary lobe hormones 8 %	
			Dermatologicals 6.9 %	
Stultz et al., 2007 [37]	USA, cohort study, *n* = 45 breastfeeding women	12 months	96 %	Quality score: Abstract: 50 %, Paper: 77 %
	Women after delivery filled out a prenatal questionnaire and were followed up monthly by telephone for 12 months postpartum or until cessation of breastfeeding or until the close of the study in Jan 2007. They were also instructed to keep a diary regarding use of medicine.		Vitamins 73 %	
			NSAIDs 71 %	Limitation(s): Very small study. ATC classification was not used. Detail breastfeeding information was not available. The impact of medicine use on breastfeeding was not studied.
			Acetaminophen 58 %	
			Progestins 24 %	
			Antimicrobials 22 %	
Bakker et al., 2006 [38]	Netherlands, cohort study, *n* = 5.412 postpartum women	3 months	68 %	Quality score: Abstract: 88 %, Paper: 68 %
	Pharmacy records of women giving birth between 1994 and 2003 were collected from the InterAction database which contains prescription drug information from community pharmacies.		Iron preparations 30.4 %	
			Systemic antibacterials 13.3 %	Limitation(s): Actual drug intake bias as data source was register. No breastfeeding information was available
			Laxatives 6.9 %	
			NSAIDs 5 %	
Schirm et al., 2004 [39]	Netherlands, cross-sectional study, *n* = 549	<6 months	66 % of 451 breastfeeding women	Quality score: Abstract: 88 %, Paper: 70 %
	Questionnaires were handed out to women with a child < 6 months through 85 Well-Baby Clinics over a 6 week period in 2002. 549 women responded and 451 of them breastfed and 297 of them used medicine.		Vitamins 40.8 %	
			Oral analgesics 36.8 %	Limitation(s): ATC classification was not used . The impact of medicine use on breastfeeding was not studied statistically.
			Antiinfectives 14.6 %	
			Gastrointestinal drugs 6.9 %	
Lamounier et al., 2003 [42]	Brazil, cross-sectional study, *n* = 2,173	Immediate postpartum period	96 %	Quality score: Abstract: 75 %, Paper: 60 %
	2,173 women giving birth in four hospitals of Belo Horizonte city in Brazil between July 1998 and July 1999 were interviewed using questionnaire. Medical records were also checked.		Anti-inflammatory 77.8 %	
			Analgesics 75.5 %	Limitation(s): ATC classification was not used. Detail breastfeeding information was not available. The impact of medicine use on breastfeeding was not studied.
			Antibiotics 17.8 %	
Jones and Brown 2003 [41] & 2000 [44]	UK, cross-sectional study, *n* = 820	Within 5 days after delivery and after hospital discharge	56.5 % within 5 days after delivery	Quality score: Abstract : 38 %, Paper: 85 %
	Questionnaires were sent to postpartum women, in southern England between March and April 1995 and 820 breastfeeding women responded.		55 % after hospital discharge	
			Antibiotics 14.27 %	Limitation(s): ATC classification was not used. The impact of medicine use on breastfeeding was not studied.
			Analgesics 3.3 %	
Olesen et al. 1999 [45, 46]	Denmark, cohort study, *n* = 15,756 to 16,001	12 weeks	34.2 % to 34.7 %	Quality score: Abstract: 88 %; Paper: 64 %
	Information about women’s prescription drug use during pregnancy and 12 weeks postpartum was collected from North Jutland Prescription Database from 1 Jan 1991 to 31 Dec 1996 and linked to Danish Medical Birth Register.		Penicillins 20.1 %	
			Opthalmologicals 15.5 %	Limitation(s): Actual drug intake bias as data source was register. No breastfeeding information was available
			Dermatological corticosteroids 5.7 %	
			(% based on total number of prescriptions)	
Thomas et al., 1994 [49]	India, cross-sectional study, *n* = 539	6 weeks	100 %	Quality score: Abstract: 38 %, Paper: 70 %
	Women who gave birth at a Southern Indian Hospital between June and Sept 1989, were interviewed using a questionnaire from the day of discharge to 6 weeks postpartum during their subsequent visits in hospital. Hospital charts were also reviewed.		Vitamins and minerals 100 %	
			Antipyretics 53.1 %	Limitation(s): ATC classification was not used. The impact of medicine use on breastfeeding was not studied.
			Anti-inflammatory 49.2 %	
			Antibiotics 37.8 %	
Uppal et al., 1993 [50]	India, cross-sectional study, *n* = 500	N/A	90 %	Quality score: Abstract: 63 %, Paper: 75 %
	200 women giving birth in Nehru Hospital, 200 attending the postpartum clinic at the same hospital, and 100 women living in a rural area were interviewed between Nov 1989 and May 1990. Hospital records were also checked.		For hospital, postpartum clinic and community settings-	
			Antibiotics: 90 %, 86 % and 13 % respectively	Limitation(s): ATC classification was not used. The impact of medicine use on breastfeeding was not studied. Very little breastfeeding information.
			Analgesics: 56 %, 70 % and 37.6 % respectively	
Blomquist and Soderman, 1991[52]	Sweden, cross-sectional study, *n* = 195	Up to 4 months	70 % (excluding vitamins)	Quality score: Abstract: 75 %, Paper: 65 %
	Women giving birth between 12 Jan and 8 Feb 1987 were asked to answer a questionnaire after hospital discharge. 195/229 women responded.		Vitamins 45 %	
			Pituitary hormones 29 %	Limitation(s): The impact of medicine use on breastfeeding was not studied. Very little breastfeeding information
			Sex hormones 18 %	
Matheson et al., 1990 [53]	Norway, retrospective survey, *n* = 885	3–5 months	69 %	Quality score: Abstract: 63 %, Paper: 70 %
	Women 3–5 months after delivery responded to a postal questionnaire in 1985.		Analgesics/antipyretics 32 %	
			Dermatologicals 19 %	Limitation(s): ATC classification was not used. The impact of medicine use onbreastfeeding was not studied.
			Antihaemorrhoidals 15 %	
Passmore et al., 1984 [55]	Ireland, cross-sectional study, *n* = 2,004	Immediate postpartum period	99 %	Quality score: Abstract: 75 %, Paper: 65 %
	Medicine charts of women giving birth in three hospitals of Belfast between July to Sept 1982.		Analgesics 78.4 %	
			Antibacterials 15.5 %	Limitation(s): ATC classification was not used. Very little breastfeeding information.
			Hypnotics 36 %

**Table 2:** Impact of maternal medicines on breastfeeding

Author and publication year	Country, study type, sample, design and settings	Results	Quality score and limitation(s)
Chaves et al., 2011 [33]	Brazil, cohort study, *n* = 246	Duration of breastfeeding was longer in women who used no medicine or who used medicines compatible with breastfeeding (*p* < 0.05).	Macfarlane’s checklist: See Table 1
	Other information is available in Table 1.		Newcastle-Ottawa: 8/9 (Selection: 4*, Comparability:2*, Outcome: 2*)
			Limitation(s): See Table 1
Lee et al., 2000 [43]	Canada, cohort study, *n* = 36 (exposure group)	44 % of women receiving PTU initiated breastfeeding compared to 83 % in the two control groups (Group 1 vs Group 2, *p* < 0.01; group 1 vs group 3, *p* < 0.01).	Macfarlane’s checklist: Abstract: 88 %, Paper: 73 %
	Women requiring propylthiouracil (PTU) during pregnancy (Jan 1990 to Sept 1997) were recruited and interviewed postpartum regarding their choice of infant feeding method. 36 women required PTU postpartum (Group 1); 30 did not (Group 2); 36 healthy women were controls (Group 3).		Newcastle-Ottawa: 8/9 (Selection: 4*, Comparability: 2*, Outcome: 2*)
			Limitation(s): Study is based on selective medicine
Ito, 1999 [47]	Canada, cohort study, *n* = 88	69 women used medicines (Group 1) and 19 women did not start the medicine of concern (Group 2). 22 (32 %) of Group 1 women stopped breastfeeding before the infant was 6 months old while 1 (5 %) of the Group 2 women did so (*p* < 0.04).	Macfarlane’s checklist: Abstract: 88 %, Paper: 82 %
	Breastfeeding women who received reassuring advice about compatibility of medicine from the Motherisk Teratogen Information Center in Toronto about their medicines in 1993 were followed up by interview up to cessation of breastfeeding or until the infant reached to 7 months.		Newcastle-Ottawa: 7/9 (Selection: 4*, Comparability: 1*Outcome: 2*)
			Limitation(s): Small sample size
Ito et al., 1995 [48]	Canada, cohort study, *n* = 34 (exposure group)	50 % of women receiving medicines started breastfeeding compared to 85 % in control group (*p* = 0.004). Median duration of breastfeeding in medicine group was significantly shorter than that in control group (4.7 ± 2.6 vs 9.3 ± 5.7 months, *p* < 0.005). 65 % of women (11/17) taking antiepileptics stopped breastfeeding within 6 months whereas in control group, only 21 % of women (6/29) did so (*p* < 0.008).	Macfarlane’s checklist: Abstract: 88 %, Paper: 86 %
	Women receiving antiepileptics during pregnancy were interviewed between April and June, 1993, by the Motherisk Teratogen Information Center in Toronto. 34 pregnant age-matched women were controls.		Newcastle-Ottawa: 7/9 (Selection: 4*, Comparability: 1*, Outcome: 2*)
			Limitation(s): Study is based on selective medicine
Ito et al., 1993 [51]	Canada, prospective cohort study, *n* = 203	125 women were followed within 32 weeks of the initial consultation. 106 women started antibiotic therapy and 7 % of them stopped breastfeeding during therapy.	Macfarlane’s checklist: Abstract: 100 %, Paper: 64 %
	Breastfeeding women who consulted the Motherisk Teratogen Information Center in Toronto between Jan 1990 and Jul 1991 about the compatability of antibiotics with breastfeeding after receiving antibiotic prescriptions.		Newcastle-Ottawa: 6/9 (Selection: 4*, Comparability: 1*, Outcome: 1*)
			Limitation(s): Study is based on selective medicine, small sample and has attrition bias

The studies were published between 1984 and 2011, and were conducted between 1982 and 2007. With the exception of 2 studies in India and 2 in Brazil, most studies were conducted in developed countries: 4 in Canada, 2 in the Netherlands, 2 in Norway, 2 in Sweden, 1 in Denmark, 1 in the UK, 1 in Ireland and 1 in the USA.

Excepting the large register-based pharmacoepidemiological studies or articles [34, 36, 38, 45, 46] (*n* = 5412 to 106,329), the sample size of most studies was small to medium (*n* = < 100 to ˂ 1000).

### Quality assessment

#### Macfarlane’s checklist score

Tables 1 and 2 show the quality scores of the 18 studies evaluated. There was a large variation in quality between the abstracts and full articles of the individual studies. Overall, the quality score for the abstracts ranged from 38 to 100 %, and for the full-text articles from 60 to 86 %.

#### Newcastle-Ottawa quality assessment score

Five cohort studies with information about the impact of maternal use of medicine on breastfeeding outcomes were eligible for assessment using the Newcastle-Ottawa quality assessment scale (Table 2). The scores ranged between 6 and 8 stars (maximum = 9 stars).

### Extent of medicine use in postpartum women and most frequently used medicines

Sixteen reports [33, 34, 36–39, 41, 42, 44–46, 49, 50, 52, 53, 55] based on 14 studies had information about different types of medicines used by women during lactation or the postpartum period, but these studies either did not have breastfeeding information or did not analyze the impact of maternal medicine use on breastfeeding outcomes statistically except for one [33]. Table 1 summarizes the extent and type of medicines used by postpartum women.

All of these studies, except one [46] suggested that more than 50 % of women required medicine during the postpartum period. However, hospital based studies indicated that the maximum extent of medicine use in women during the postpartum period could be up to 100 % if vitamins/minerals are included [49], while large register-based pharmacoepidemiological studies reported maximum use up to 57 % [36] without any breastfeeding information.

The most commonly used medicines in register-based studies were systemic antibacterials, if sex hormones (e.g. oral contraceptive) were not considered. In the rest of the studies, analgesics/antipyretics, nonsteroidal anti-inflammatory drugs (NSAIDs) and antibacterials/antibiotics were the most commonly used medicines if vitamins, minerals, or iron preparations were not considered.

### Comparison of types of medicine used by postpartum women in different countries according to ATC classification system

Large pharmacoepidemiological articles [34, 36, 38, 45, 46] and one survey [52] used the ATC classification system to report medicine use in women during the postpartum period. Systemic antibacterials (ATC code J01) were commonly used short-term medicines according to information available in large pharmacoepidemiological studies conducted in Sweden [34], Norway [36], the Netherlands [38] and Denmark [46] ranging between 13 and 16 % of medicines used (Table 1). Table 3 depicts the proportion of postpartum women who used medicines for different chronic diseases in Sweden, Norway and the Netherlands.

**Table 3:** Proportion of women using medicines for various chronic diseases in the first three months postpartum

Medication group	Proportion of women (%)		
	Sweden [34] *n* = 102,995	Norway [36] *n* = 106,329	Netherlands [38] *n* = 5,412
Cardiovascular drugs (C)	3.06	4.9	N/A
Thyroid therapy (H03)	1.81	1.4	1.0
Antiasthmatics (R03)	1.51	1.3	2.1
Antidepressants (N06A)	1.6	0.7	2.1^a^
Drugs for diabetes (A10)	0.4	0.3	0.4
Antiepileptics (N03)	0.3	0.3	0.3

^a^Antidepressants including antipsychotics (N05A)

### The impact of postpartum use of maternal medicine on breastfeeding outcomes

Five studies [33, 43, 47, 48, 51] analysed the impact of maternal medicine (group/specific medicine) on breastfeeding (Table 2). One study by Chaves et al. from Brazil [33] showed the relationship between maternal use of medicines and duration of breastfeeding classifying medicines according to Hale’s *Medications and Mothers’ Milk* 2004 [56] and by the American Academy of Pediatrics 2001 publication on ‘Transfer of drugs and other chemicals into human milk’ [17]. The authors concluded that women using unclassified drugs according to Hale’s criteria were most likely to cease breastfeeding compared to women using breastfeeding compatible drugs. A study by Lee et al. (2004) showed that the majority of women (57 %) receiving propylthiouracil (a medicine for hyperthyroidism) did not initiate breastfeeding [43] and a study by Ito (1999) indicated that a significant proportion of women (32 %) taking a medicine of concern terminated breastfeeding earlier than women not taking a medicine of concern (*p* < 0.04) [47]. Ito et al. (1995) in an earlier study of antiepileptics also showed that women receiving antiepileptic medicines had both lower initiation and duration of breastfeeding compared to women receiving no antiepileptics postpartum [48]. In addition, Ito et al. (1993) in a study of antibiotics showed that 7 % of women stopped breastfeeding during antibiotic therapy [51].

### Women’s concerns, behaviour and decision-making about use of medicines while breastfeeding

Many women hesitate to combine medicine use with breastfeeding, or do not initiate breastfeeding, or stop taking medicines while breastfeeding, or quit breastfeeding, or chose formula feeding while taking medicines [39, 43, 47, 48, 51] (Table 4). One study found that many women were more doubtful about medicine use during lactation than during pregnancy [53].

**Table 4:** Women’s concerns and behaviour towards use of medicines while breastfeeding

Authors and year of publication	Women’s concerns and behaviour towards use of medicines while breastfeeding
Schirm et al., 2004, Netherlands [39]	297 women used medicine. 30 % of them hesitated to take a medicine while breastfeeding. Almost 10 % of 297 women (breastfeeding yes, medicine yes) stopped either breastfeeding or medicine use. 17 % of 154 women (breastfeeding yes, medicine no) indicated they would have used medicine if they were not breastfeeding. About 12 % of 78 women (breastfeeding no, medicine yes) mentioned medicine use as the reason for not breastfeeding.
A cross-sectional survey of postpartum women about their medicine use and breastfeeding	
*n* = 549	
Lee et al., 2000, Canada [43]	60 % of 20 formula feeding women mentioned that physicians’ advice or their concern about the medicine was the primary reason for not breastfeeding. Women given advice by their physician in favor of breastfeeding were more likely to breastfeed than women not given this advice (Relative Risk: 5.48; 95 % CI: 1.28–23.40).
A cohort study of women requiring propylthiouracil	
*n* = 36 (exposure group)	
Ito, 1999, Canada [47]	19 women (22 %) did not start the medicine of concern while breastfeeding despite their need for medicine.
A cohort study of medicine use in breastfeeding women who had concern for their medicines	
*n* = 88	
Ito et al., 1995, Canada [48]	50 % (17/34) of women did not initiate breastfeeding and chose to formula feed and 88 % of them mentioned medicine was the reason of formula feeding.
A cohort study of antiepileptics	
*n* = 34 (exposure group)	
Ito et al., 1993, Canada [51]	125 (62 %) women were followed within 32 weeks. 19 (15 %) women did not initiate the antibiotic therapy. 21 % women either did not start the required medicine or stopped breastfeeding while taking medicine.
A cohort study of antibiotic use and breastfeeding	
*n* = 203	
Matheson et al., 1990, Norway [53]	17 % women showed more doubts about medicine use during lactation than during pregnancy. 33 % had similar risk perception about medicine use in pregnancy and lactation.
A survey of medicine use in postpartum women	
*n* = 885	

### Critical findings

This systematic review summarizes the methodological quality, design and findings of eighteen observational studies about postpartum women’s use of medicines with or without breastfeeding information. Findings suggest that medicine use in postpartum women is very common, but due to differences in study design, inconsistent reporting and large variations in sample sizes (*n* = < 100 to 106,329), the prevalence of medicine use by postpartum women in different countries are not comparable. Moreover, all large pharmacoepidemiological, register-based studies or articles  [34, 36, 38, 45, 46] were limited by lack of breastfeeding information and may be biased as registers do not contain information about whether or not individuals actually used the medicine. In addition, these studies were mainly focused on medicine use during pregnancy and medicine use information is available for only up to 3 months postpartum. On the other hand, cross-sectional surveys or small cohort studies with little breastfeeding information do not represent the diverse range of medicines used by women especially for chronic illnesses such as depression, asthma, hypertension, thyroid problems, diabetes and epilepsy.

Although five studies indicated that maternal use of certain medicines was associated with lower initiation and/or duration of breastfeeding [33, 43, 47, 48, 51], we cannot claim that all medicine use has a negative impact on women’s breastfeeding practices. Moreover, none of these studies received a full score in the quality assessment, and are not free from bias. The study by Ito et al. (1993) [51] might have attrition bias as outcome data was only available for 62 % of the women (125 of 203). Two other studies [33, 47] were based on maternal use of different types of medicines, they were limited by their small sample size (*n* = 246 and 88 respectively) and neither study analysed the impact of the specific drug/group according to acute or chronic use or type of illness.

### Strengths and limitations

The major strength of this review is the use of a systematic approach. This is the first comprehensive review focusing on extent of medicine use in postpartum women and impact of maternal medicine use postpartum on breastfeeding. The methodological qualities of the included studies were also assessed using quality appraisal tools.

The main limitation of this review is that we were unable to conduct a meta-analysis because of the wide differences in individual study design and outcome measures. Prescription and non-prescription medicine use were not differentiated in the included studies, and this is also a limitation. We did not include a complete search for every therapeutic group and medicine as search terms.

## Conclusion

The review of 18 studies found several studies including maternal use of medicines for certain chronic diseases (e.g. epilepsy, hyperthyroidism) demonstrated a negative impact on initiation and /or duration of breastfeeding. Further study is warranted as these studies were conducted more than a decade ago. Moreover, we did not get a complete picture why women discontinued breastfeeding or did not start. Was this due to health professionals’ advice or other difficulties faced by women? This review also reveals the need for a uniform reporting system of medicine use in breastfeeding or postpartum women using WHO’s ATC classification system. As the existing register-based, large scale studies are limited with data only up to 3 months postpartum and have no breastfeeding information, more research is needed both in developing and developed countries to establish baseline information regarding medicine use in breastfeeding women. Qualitative studies are also required to understand how postpartum women make their decisions about breastfeeding and medicine use and what influences their decision-making. Improved understanding about maternal medicines and breastfeeding could lead to improved evidence-based practice, and thus reduce the dilemma of maternal medicines and breastfeeding.

## Additional files

Additional file 1:
**Quality assessment checklist for observational studies adopted by Macfarlane et al. [** 26 **] from Downs and Black [** 27 **] and Crombie [** 28 **].** (DOCX 19 kb) Additional file 2:
**Newcastle-Ottawa quality assessment scale for cohort studies [** 29 **].** (DOCX 16 kb)
