# Supplementary material for: Postpartum women’s use of medicines and breastfeeding practices: a systematic review
Source: Int Breastfeed J. 2015 Oct 28;10:28. doi: 10.1186/s13006-015-0053-6 (PMC4625926; doi:10.1186/s13006-015-0053-6)
Supplement: Additional file 1: — Quality assessment checklist for observational studies adopted by Macfarlane et al. [ 26 ] from Downs and Black [ 27 ] and Crombie [ 28 ]. (DOCX 19 kb) [file 13006_2015_53_MOESM1_ESM.docx]

Additional file 1

**Quality assessment checklist for observational studies adopted by Macfarlane et al [**[**26**](#_ENREF_26)**] from Downs and Black [**[**27**](#_ENREF_27)**] and Crombie [**[**28**](#_ENREF_28)**].**

| **Items for quality assessment** |
| --- |
| **Abstract** |
| 1. Is the hypothesis/aim/objective of the study clearly described? |
| 1. Is the design of the study described? |
| 1. Is the source of the subjects studied stated? |
| 1. Is the sample size stated? |
| 1. Is the participation/follow up rate stated? |
| 1. Are the outcomes of interest described? |
| 1. Are any results given? |
| 1. Are any conclusion stated? |
|  |
| **Paper** |
| 1. Is the hypothesis/aim/objective of the study clearly described? |
| 1. Are the main outcomes to be measured clearly described in the introduction or Methods section? |
| 1. Is the design of the study described? |
| 1. Is the setting of the study described? |
| 1. Is the source of the subjects studied stated? |
| 1. Is the distribution of the study population by age and gender described? |
| 1. Is the sample size stated? |
| 1. Is the participation/follow up rate stated? |
| 1. Are nonparticipants/subjects lost to follow up described? |
| 1. Do the authors describe the effort to increase the participation/follow up rate? |
| 1. Are the main findings of the study clearly described? |
| 1. Are the statistical methods described? |
| 1. Have actual probability values been reported (e.g. 0.035 rather than ˂0.05 ) for the main outcomes except   where the probability value is less than 0.001)? |
| 1. Are confidence intervals given? |
| 1. Are any conclusions stated? |
| 1. Were the subjects asked to participate in the study representative of the entire population from which   they were recruited? |
| 1. Were those subjects who were prepared to participate representative of the entire population from which   they were recruited? |
| 1. Was the participation/follow up rate ˃ 80% |
| 1. Were the main outcome measures used accurate (valid and reliable)? |
| 1. Was the sample size justified? |
| 1. Was the follow up of cohorts adequate? (Cohort only) |
| 1. Analysis adjusts for length of follow-up? (Cohort only) |
|  |
